# Individual vs. Team Sport Failure—Similarities, Differences, and Current Developments

**DOI:** 10.3389/fpsyg.2022.930025

**Published:** 2022-06-24

**Authors:** V. Vanessa Wergin, Clifford J. Mallett, Jürgen Beckmann

**Affiliations:** ^1^Faculty of Health and Behavioural Sciences, School of Human Movement and Nutrition Sciences, The University of Queensland, St Lucia, QLD, Australia; ^2^Faculty of Sport and Health Sciences, Technical University of Munich, Munich, Germany

**Keywords:** choking under pressure, performing under pressure, collective team collapse, team dynamics, team choking

## Abstract

The construct of “choking under pressure” is concerned with the phenomenon of unexpected, sudden, and significant declines in individual athletes’ performances in important situations and has received empirical attention in the field of sport psychology. Although a number of theories about the reasons for the occurrence of choking under pressure exist and several intervention approaches have been developed, underlying mechanisms of choking are still under debate and the effectiveness of existing interventions remains contested. These sudden performance declines also occur in team sport. “Collective sport team collapse,” which describes the situation when an entire sport team underperforms significantly within an important competitive situation, has received less empirical attention, in comparison to individual choking research. While there are a few studies that have investigated causes of collective team collapse, understandably, there has been limited empirical investigation of preventative and intervention strategies. Although the two constructs appear to share several similar characteristics and mechanisms, research has not yet examined the conceptual, theoretical, empirical, and practical links between choking under pressure and collective sport team collapse. In this review article, we seek to examine these similarities and differences and identify new ways of thinking about future interventions. Furthermore, current empirical understandings in the field of choking under pressure and collective sport team collapse are presented and the most effective intervention approaches for both constructs are introduced. On the basis of this examination, we modestly make some initial recommendations for sport psychological practitioners and future research.

## The Constructs of Choking Under Pressure and Collective Team Collapse

Performing when it matters the most is certainly important to athletes and may not only determine victory or defeat, but impact athletes’ careers, like the one of golfer Jean Van de Velde, whose dramatic underperformance in the final round of The Open Championship is ranked among the top performance failures in sport history (Siegel, 2011). While some athletes perform to their expected standards or on rare occasions surpass previous expectations, others tend to show impaired performances in pressure situations, which usually include important competitions and/or the presence of a large audience (e.g., Wallace et al., 2005). When professional track and field athletes stumble in the Olympics or elite soccer players miss the game deciding penalty shot in the finals of the World Championships, they likely experience “choking under pressure”. Initially defined by Baumeister (1984) as “performance decrements under circumstances that increase the importance of good or improved performance” (p. 610), several definitions of choking under pressure have developed; however, no global definition exists (Buszard et al., 2013; Jackson, 2013; Mesagno and Hill, 2013).

Individual athletes choking under pressure has been of interest to researchers, pracitioners, and the public, but teams (as a collective) have also suddenly and significantly underperformed in important games. This frequently takes the form of a sudden collapse of the performance and has therefore been described as “team collapse” (Apitzsch, 2006; Wergin et al., 2018). A famous example of collective team collapse is the 1–7 loss of the Brazilian team against Germany in the semi-finals of the 2014 FIFA World Cup in front of their home audience (Filho, 2021). An applied analysis showed that Brazil started the game with a regular performance, which was disrupted by the first goal scored by Germany, leading to a series of further four goals scored in the next 15 min of the game (Filho, 2021). Even though the similarities between individual choking and team collapse might appear to be obvious, surprisingly, the two strands of research have remained relatively unconnected to date. The present article attempts to integrate research on choking under pressure and collective team collapse to identify mutual characteristics to inform future interventions.

Since both constructs are based on different literature, we first present research on choking under pressure in individual sports as well as on existing interventions. Thereafter we introduce literature on collective team collapse and potential interventions. Finally, we discuss links and differences between the two constructs to advance insights and develop interventions to circumvent performance failure on both the individual and team level.

## Choking Under Pressure

Research on choking under pressure has largely focused on explaining the phenomenon of dramatic underperformances in pressure situations and their underlying mechanisms. Generally, literature distinguishes between attention-based models and self-presentation models of choking under pressure (e.g., Mesagno et al., 2011; Mesagno and Beckmann, 2017), which are outlined in the following sections.

### Attention-Based Models of Choking Under Pressure

Despite the lack of a consensus in definition, performance anxiety appears to play a key role in causing athletes to underperform or “choke” under pressure (e.g., Mesagno and Hill, 2013; Mesagno and Beckmann, 2017) and has been referred to in several theories. According to attention-based theories of choking under pressure, anxiety in important performative situations leads the athlete to either focus so intensely on the movement that the movement cannot be executed regularly anymore (i.e., increased self-focus) or evokes distracting thoughts in the athlete, causing a lack of attention (i.e., distraction) (Baumeister, 1984; Beilock and Carr, 2001). An increased self-focus is typically accompanied by a shift in focus from sport relevant information to internal cues, such as the execution of the movement or an inner feeling of the body. The athlete may closely monitor the movement execution (Beilock and Carr, 2001) or try to consciously control certain movement components (Masters, 1992), equally leading to a disruption of the movement and causing choking under pressure.

Distraction, on the other hand, is characterized by interrupting myriad thoughts, which are not related to the competitive situation and might, for example, be related to possible outcomes of the competition or past experiences in previous tournaments (Eysenck et al., 2007; Oudejans et al., 2011; Englert and Oudejans, 2014). Eysenck and Calvo (1992) argue that this is likely due to an increase in anxiety during the performance situation, leading to an increased activity in the working memory, which reduces the capacity to focus on task-relevant stimuli. A further explanation is that anxiety increases athletes’ attention to threat-related stimuli, such as the consequences of a possible failure (Eysenck et al., 2007), which not only potentially shifts an athlete’s attention but also might contribute to additional anxiety.

### Self-Presentation Models of Choking Under Pressure

Theories based on self-presentation as main cause of choking assume that we are always aiming at presenting ourselves to others in a favorable way, creating desirable impressions, and prohibiting the development of undesirable impressions (Leary and Kowalski, 1990; Leary, 1992). This is especially true for athletes with a strong need to be perceived as skilled and talented by coaches and audience, as this may have a positive impact on selection decisions and therefore on their career (Prapavessis et al., 2004). The striving for an ideal self-presentation does, however, have the potential to increase anxiety in athletes (Wilson and Eklund, 1998) and lead to choking in important performance situations. Mesagno et al. (2011) argue that certain personality traits increase this anxiety about self-presentation in athletes. An athlete who is worried about the judgment of coaches or audience, for example, is likely experiencing higher levels of anxiety and a higher susceptibility to choking under pressure. In accordance with this, self-confidence and self-consciousness are further crucial factors to consider when performing in front of an audience (Wang et al., 2004).

## Current Developments and Interventions in Choking

Several prevention and intervention strategies for choking under pressure have been developed. As anxiety has been shown to play a major role in causing choking under pressure (Mesagno and Beckmann, 2017), many of the strategies focus on reducing the feeling of anxiety or its outcomes, such as attentional problems or an increase in arousal. Hence, we present the following most popular interventions for choking under pressure in regard to their intended goal of attentional regulation, change of appraisal, or arousal regulation, which is in line with Gross’ (2015) initiation for emotion regulation.

### Attention Regulation

Building upon the assumptions of self-focus models, interventions aiming at reduced conscious control and a reduced application of explicit knowledge have been developed. Masters (2000) used an analogous motor learning approach to limit explicit knowledge and foster the use of implicit knowledge in athletes, which applies biomechanical metaphors to teach complex actions like, for example, drawing a triangle with the bat for a correct execution of a top spin hit in table tennis. In several experiments, Masters (2000) showed that the performance of individuals who had applied the implicit learning strategy was more resilient to stress and pressure than the performance of individuals who had applied explicit learning. Similar results were achieved by Liao and Masters (2001) in table tennis players and by Lam et al. (2009) in basketball players. These experiments provide an approach allowing athletes to recall their performance in an automated and unconscious way, making them less susceptible to choking under pressure.

Contrary to struggling with an increased self-focus, athletes may experience difficulties focusing on the task and feel distracted by task-irrelevant thoughts. An intervention that has shown to help athletes adjust their focus are pre-performance routines. These routines mainly aim at helping athletes focus on the task, prohibiting a distraction by undesired thoughts (Mesagno and Beckmann, 2017). Pre-performance routines typically consist of a sequence of thoughts and actions (e.g., cognitive preparation, deep breathing, or cue words) directed toward the task to be performed and have been shown to stabilizes athletes’ performance in pressure situations (e.g., Mesagno and Mullane-Grant, 2010).

Pre-performance routines can easily be combined with strategic self-talk (Galanis et al., 2021), which may further involve an application of rhythms and/or music. German basketball player Dirk Nowitzki for example acknowledged that he hummed David Hasselhoff’s song “Looking for Freedom” to get himself ready for conducting a free-throw (Pollakoff, 2015). This technique has been scientifically investigated and shown to be especially effective when the music matched the characteristics of the movement (e.g., Pates et al., 2003; Mesagno et al., 2009). Another technique to be applied prior to the execution of performance is quiet eye (Vickers, 2007), where an athlete visually fixates a target prior to executing the movement. Quiet eye can be combined with other pre-performance routines such as breathing or thinking (Mesagno et al., 2019).

A further technique that has shown to support athletes to enter a mental state of automatism is imagery or visualization. Imagining the movement prior to its conduction has been shown to protect athletes from choking (e.g., Hill et al., 2011; Hill and Shaw, 2013). The imagery task should be adjusted to the skill level of the athlete, for example by having novice athletes imagine the movement from a third-person perspective, which is easier, while more advanced athletes may use the more effective but also more difficult first-person perspective (Krawietz, 2013).

### Change in Appraisal

As discussed above, choking under pressure and unhelpful emotions, such as athlete anxiety, go hand in hand, as anxiety usually drives underperformance in important sports situations (Mesagno and Beckmann, 2017). It is therefore unsurprising, that several intervention strategies have targeted unhelpful emotions and include emotion regulation strategies. One of these strategies is cognitive reappraisal, involving a change of the evaluation of the negative stimulus or the situation that initially caused the emotion (Gross, 2002). An athlete could for example evaluate the competitive situation as a chance to play some good sport and show his or her own skills rather than perceiving it as a threat of potentially losing the game. A further variation of this technique is cognitive reframing, which refers to the identification of negative thoughts and a goal-oriented restructuring of these thoughts into positive ones. Cognitive reappraisal has been shown to help athletes cope with pressure (Balk et al., 2013).

Supplementary to re-evaluating the situation or reframing thoughts, athletes may use the strategy of relativization of the situation (Jermann et al., 2006). This strategy aims at putting the current pressure situation into a broader perspective. A tennis match can be perceived as a threat for the individual player, but compared to other, rationally more threatening situations, it is perceived as less significant. Within this strategy, watching news and putting the own sport in the perspective of being “just a sport” are common tactics.

### Arousal Regulation

Apart from interventions addressing attention and emotion, a variety of interventions aiming at the regulation of athletes’ arousal have been developed. One of the best-established interventions targeting the downregulation of athletes’ arousal is resilience or simulation training. This training technique is often applied by coaches and typically includes a pressure or anxiety induction during practice in order to get athletes used to a certain level of stress and increase their resilience to this stress (e.g., Beilock and Carr, 2001; Oudejans and Pijpers, 2009, 2010; Mesagno et al., 2015; Fletcher and Sarkar, 2016).

Further strategies that similarly target a downregulation of arousal in athletes are typical relaxation methods, including for example deep breathing, mindfulness, or hypnotic practices (e.g., Parnabas et al., 2014; Scott-Hamilton et al., 2016). While the original versions of these techniques are rather difficult to apply in sports, several adaptions have been made, essentially shortening the techniques to allow for an application in the field. Breathing techniques usually focus on a prolonged exhalation and can easily be adapted to sports, for example, by letting athletes count during their breathing and increasing the amount of counted numbers during the exhaling phase. Breathing techniques can further be combined with mindfulness practices, which add a focus on other bodily sensations or on visual and auditive stimuli to the routine.

A further technique that has been shown to effectively reduce anxiety and other unhelpful emotions, and therefore may be used to interfere with choking, is embodiment. Embodiment aims at changing the mental state of an athlete through body posture, physical expression, and body tension (Gallagher, 2005). The technique is based on the principles of muscles sending information about posture back to the brain, influencing feelings, information processing, and motivation. Since athletes can consciously control their body posture, embodiment can be applied as a tool to make oneself feel less anxious and more self-confident. Another embodiment technique is the left-hand dynamic handgrip (Beckmann et al., 2013). Using this approach, right-handed athletes clench their left hand dynamically for 10–15 s, which has been shown to produce a state of cortical relaxation (Cross-Villasana et al., 2015), reducing anxiety and the experience of choking under pressure in athletes (e.g., Beckmann et al., 2013, 2021).

Taken together, existing research on choking under pressure has mainly focused on self-presentation and attention-based models and designed interventions tailored to those theories. We illustrated existing interventions in the light of Gross’ (2015) theory of emotion regulation, as all of them target different stages of the emotion development process, indicating that a holistic approach to emotion regulation may be key to the development of intervention strategies.

## Collective Sport Team Collapse

Although it is assumed most team sport athletes are familiar with the phenomenon of an entire team suddenly underperforming simultaneously, it empirically remains poorly understood. Similar to choking under pressure, collective sport team collapse appears to happen during important games or tournaments, when the perceived pressure on the team is high (Apitzsch, 2006) and has been defined as “a sudden, collective, and extreme underperformance of a team within a competition, which is triggered by a critical situation that interferes with the team’s interplay, a loss of control of the game, and ultimately the inability of the team to regain their previous performance level within the game” (Wergin et al., 2018, p. 5). Initial studies on collective sport team collapse showed that rather than being evoked by single causes, team collapse can be understood as a process and occurs as a result of a cascade of causes, whereby a critical event on the court typically triggers the collapse (Wergin et al., 2018, 2019).

While certain antecedents, such as a lack of attention, overconfidence about winning the game, and/or an insufficient level of experience or preparation may function as risk factors increasing the likelihood of a team collapse, the occurrence of a critical event on court can be seen as the starting point of the collapse. Such critical events may include an accumulation of mistakes in the own team, points scored by the opponent team, game interruptions (e.g., due to injury of a player), key players of the team suddenly choking, or referee decisions made against the team. These critical events change dynamics within the team on an emotional, cognitive, and behavioral level.

On an individual cognitive level, major impacts of the critical event include the perception of increased pressure, an insecurity about the situation, and despair in a way that athletes do not know what to do (Wergin et al., 2018). Furthermore, Wergin et al. (2019) found players to switch from a goal-oriented thinking to a defensive and prevention-oriented thinking, including worrying about failing the expectations that others held about their team’s performance. On a team level, athletes suffer from a perceived lack of accountability among team members. Players refuse to take responsibility for game situations (e.g., win the ball), as they do not want to be held responsible for the failure of the team (Wergin et al., 2018). They also perceive team members to individualize and to be playing on their own rather than as a collective, potentially leading to an actionist atmosphere within the team, causing individual players to try to score by physical force (Wergin et al., 2019).

Examining the emotional changes in players and the team, an increase in unhelpful emotions, especially in anxiety and anger (Wergin et al., 2018) as well as frustration (Wergin et al., 2019) can be observed in players. Interestingly, these emotions transfer from one player to another (Wergin et al., 2018, 2019), a process that has been referred to as emotional contagion (Hatfield et al., 1994) and that has been investigated in a number of studies in sport (e.g., Totterdell, 2000; Moll et al., 2010) as well as in organizational settings (e.g., Kelly and Barsade, 2001; Barsade, 2002). It can thus occur, that a player experiences the mood from a teammate without having participated in the initial emotion evoking game situation. Through this process, negative atmospheres can spread rapidly within a team facing a collective team collapse.

Changes on a behavioral level include decreased performance as well as either a cautious or a more hectic play of individual team members (Wergin et al., 2018). Furthermore, an immobility or “freezing” of players on the court has been observed as a result of increased levels of anxiety (Wergin et al., 2019). On a team level, behavioral outcomes of the collapse can become even more severe. Communication between team members decreases significantly, while externalizing blame to other team members increases (Wergin et al., 2018), eventually leading to a collapse of the main strategic system of play. The mistakes and failures occurring on an individual level appear to also transfer between team members, causing an accumulation of mistakes in the team (Wergin et al., 2018, 2019).

These changes in a team’s dynamics evoke further failure by the players of the team, again impacting emotions, cognition, and behavior, which maintains the underperformance of the team in a vicious cycle that seems difficult to arrest. Hereby, especially social factors, involving processes between teammates, such as the transfer of unhelpful emotions from one player to another, appear to hinder the team from recovering their performance and constitute a unique facet of collective team collapse.

## Current Developments and Potential Interventions in Team Collapse

Wergin et al. (2020) were able to initially record collective team collapse events in field hockey teams using GPS trackers. They found teams who indicated to have suffered from a team collapse during their games to be running significantly less in these team collapse games compared to games they had lost without experiencing a team collapse. Furthermore, Wergin and colleagues showed that negative affect increased significantly in teams experiencing a team collapse compared to the same teams losing a game without collapsing. One may assume that positive affect would similarly decrease significantly in team collapse games compared to lost games, but this was not the case. Especially negative affect appeared to impact athletes’ performance and it was assumed that, in accordance with the findings of Wergin et al. (2018), negative affect is associated with negative thoughts in athletes and prohibits them from returning to their initial performance level. Besides causing rumination, the regulation of negative affect likely affects performance as well. A relationship between affect regulation and performance has been shown in various studies (e.g., Muraven et al., 1998; Schmeichel et al., 2003; Wagstaff, 2014; Haehl et al., 2022). Wagstaff (2014) for example showed that a cycling time trial was performed worse by participants who had to regulate their affect prior to their trial compared to a control group.

Building upon this assumption that negative affect and its regulation foster the ongoing underperformance of a team, more effective affect regulation strategies may be key to the development of prevention and intervention strategies. *De-personalization* is a concept within the Social Identity Theory (Tajfel, 1978; Tajfel and Turner, 1979) that suggests that when our sense of self is fully immersed in the social group or team, we behave prototypically consistent with the group and share a social identity (“we-ness”), whereby we derive our self-concept from the group. The identification with the group impacts our affect and behavior, as the group takes on affective significance for us (Tajfel, 1978). Group based emotions are experienced by the individuals within the group, who now act as group members rather than as individuals (Goldenberg et al., 2016). Additionally, members of the group can experience collective emotions simultaneously, when encountering a specific event together, like for example competitions or games. These collective emotions are a form of group-based emotions and can be understood as a “synchronous convergence in affective responding across individuals” (von Scheve and Ismer, 2013, p. 406). Specifically, winning and losing constitute ultimate stressors for sports teams, evoking individual as well as collective affect in response (Tamminen et al., 2016b). Critical events, posing the beginning of the actual collapse, can be understood as similar situations, in which teams have to deal with problems and are similarly associated with a loss of the game. Such critical situations can therefore evoke negative affect and a transfer of this affect between team members (Wergin et al., 2018, 2019).

Thus, individual as well as interpersonal affect regulation strategies could support a team in enhancing its performance, when facing negative (collective) affect and affect transfer processes in a team collapse situation. Affect regulation refers to “attempts to influence emotions, in ourselves and others” (McRae and Gross, 2020) and typically relates to performance (Wagstaff, 2014). Sport psychological research has, however, mainly investigated individual strategies of affect regulation. Niven (2017) provides a conceptual framework of affect regulation that takes the possibility of interpersonal affect regulation into account. Within this framework she differentiates between intrinsic and extrinsic affect-improving and affect-worsening regulation. While intrinsic strategies to improve one’s own affect can for example consist of positive thinking or seeking social support, intrinsic affect-worsening strategies could include negative thinking or cynicism. Strategies aiming at improving others’ affect are listening to them and their problems and providing helpful advice. Strategies to worsen others’ affect could be acting annoyed or pointing out their deficits. Niven’s (2017) framework has been associated with athletes’ autonomous motivation and commitment (Tamminen et al., 2016a) as well as with team anxiety and goal achievement (Tamminen et al., 2021) and team success (Tamminen et al., 2019). As to date, no prevention or intervention strategies to counter collective sport team collapse have been invented, individual and interpersonal affect regulation and potentially a combination of both present a promising approach for future research and practice aiming at preventing collective team collapse.

In sum, research on collective team collapse indicates the importance of emotion regulation on both the individual and the team level. Intervention strategies targeting individual and interpersonal emotion regulation could be useful in stabilizing performance of individual athletes as well as their collective team performance.

## Relation Between Choking and Team Collapse

The mechanisms underlying choking under pressure and collective team collapse as well as the interventions developed to date show both differences and similarities between the two constructs. Existing research has investigated choking as an underperformance of individual athletes at a certain point in time, while collective team collapse has been investigated from a broader perspective, whereby causes and mechanisms have been described from a broader perspective as well.

However, the two main characteristics of choking, which comprise the severe underperformance of an athlete in an important, competitive situation, carrying a significant amount of pressure, are similarly part of collective team collapse. Individual choking can occur as part of the team collapse, for example, when a key player chokes, and pressure has been found to be both an antecedent of team collapse as well as an outcome that maintains the team’s underperformance, as a team usually experiences increased levels of pressure after falling behind due to a performance collapse (Wergin et al., 2018, 2019).

Furthermore, some of the specific mechanisms underlying choking under pressure have been identified in collective team collapse as well. The attentional issues that athletes typically report when facing a choking situation (e.g., Baumeister, 1984; Masters, 1992; Beilock and Carr, 2001) have also been found in collective team collapse (Apitzsch, 2006; Wergin et al., 2018, 2019), where they appear in the form of an antecedent. These attentional antecedents are mainly characterized by a lack of attention of teammates, which is similar to the distraction theory in choking literature, while an increased self-focus has not been related to team collapse so far.

Beyond that, and in accordance with self-presentation theory (Leary and Kowalski, 1990; Leary, 1992), both constructs are characterized by a fear of failure and a fear of negative evaluation by others. Anxiety, which has been described as a detrimental factor for performance recall (e.g., Kleine, 1990; Smith and Smoll, 1990; Mesagno and Beckmann, 2017; Ehrlenspiel and Mesagno, 2021) has similarly been shown to play an important role in driving collective sport team collapse (Wergin et al., 2018, 2019). While this anxiety often leads to a direct performance decrease in individual athletes as well as to a further increased anxiety level after the failure, fear of failure in sports teams can be contagious within a team. Anxiety in team sports has been shown to impact the team in terms of player responsibility or lack thereof. Specifically, there seems to be a reluctance and aversion to take responsibility for addressing the collapse, because no player wants to be held responsible for the team losing the game. This typically leads to a cautious playing behavior of team members, which makes it even easier for the opponent to score (Wergin et al., 2018, 2019).

Apart from anxiety, other negative emotions and cognitions cause players to get caught in the vicious cycle of underperformance and decreases their chances to recover their performance. In particular, the feelings of anger and frustration play major roles in choking under pressure (e.g., Gucciardi et al., 2010) and team collapse (Apitzsch, 2006; Wergin et al., 2018, 2019, 2020). When athletes experience these emotions, they often and subsequently feel desperate and overwhelmed, which often causes a conscious or unconscious surrender of the game. Within a team these emotions can easily accumulate and make the situation spiral downward further.

At first sight, collective sport team collapse may appear like a team form of choking under pressure, as many of the individual processes fostering choking can also be found in team players during a team collapse event. However, choking and team collapse differ in many other processes and mechanisms. Wergin et al. (2018) argue that due to the many social processes that are involved in the establishment of a team collapse, such as the transfer of negative emotions and cognitions and the interactions between players (e.g., blaming each other for failure), there is more to team collapse than the simultaneous choking of various players at the same time. This assumption is supported by the fact that the choking of one or more individual players in a team does not necessarily cause a team collapse. It appears to be crucial for the development of a team collapse, who the choking players are and in which situation they choke (Wergin et al., 2018, 2019). It may occur that one individual player choking, especially a key player, causes failure and choking in other players, but this social process of performance contagion that is accompanied by the transfer of emotions and behaviors, is unique to collective team collapse. The most interesting question is, which specific mechanisms are leading to these emotional, cognitive, and behavioral transfer processes, as those mechanisms can be understood as the engine keeping a team collapse running. Thus, gaining a better understanding of these processes is key to the development of prevention and intervention strategies.

Even though choking under pressure and team collapse can be understood as constructs describing two distinct phenomena and have been investigated independently to date, there are some factors, which should be considered in the promotion of future research. Research on choking under pressure could benefit from addressing determining factors that emerged in the research on team collapse. In research on choking under pressure, the view of choking as a process, including a sequence of causes or triggers, has largely been neglected. While initial theories of choking under pressure, such as self-presentation theory or self-focus and distraction theory, may well explain the underperformance in sports where a single task is performed, such as shooting sports or jumps in gymnastics, existing theories do not consider the continuous choking of an athlete throughout performances consisting of multiple tasks, performed over a longer period of time, like for example in individual racket sports (e.g., tennis, badminton, table tennis) or choreography-based sports (e.g., dancing, figure skating, rhythmic gymnastics). In these sports, where a variety of tasks are performed for a certain duration, choking under pressure may occur in form of a dynamic self-reinforcing process. Initial underperformances can evoke further negative thoughts and emotions, which again impact performance and create even more pressure due to the initial underperformance. Choking in these sports can be understood as a process similar to collective team collapse. Therefore, some of the underlying mechanisms of collective team collapse could also play a role in fostering choking under pressure in individual sports.

Furthermore, although individual sports are performed by one person, social factors relating to other persons surrounding the athlete and their impact on the athlete, like in team collapse situations, might also be considered. While social interactions in team collapse situations, such as blaming each other for failure, mainly occur between players of a team, coaches, the audience, opponents, and potentially parents of youth athletes may exert a similar influence on athletes performing individual sports. An example may be provided by athletes choking when their coach enters the competition arena. Choking in this case is likely triggered by social interactions between coach and athlete during both practices and competitions. Also, system characteristics are worthy of investigation to examine social communications, such as finger pointing and increasing blaming behaviors from spectators and coaches.

Additionally, it should be remembered that athletes in many individual sports also have teammates. Even though they may not always perform simultaneously, but after another, like for example in relay sports, interpersonal behaviors of these teammates matter. It is equally important for individual athletes, that their teammates support them and interact with them in a positive way when facing a challenging situation. While showing negative emotions or behaviors toward a teammate in relay sports may not produce a team collapse, negative interactions, such as finger pointing, may cause an individual performer to choke.

Therefore, considering both choking mechanisms in team collapse as well as team collapse mechanisms in choking is important for gaining a better understanding of both constructs and being able to intervene on both the individual and team level. Integrating knowledge we have of both constructs, like for example viewing choking from a process perspective, can support the development of new holistic interventions.

## Practical Implications

It is important for coaches and sport psychological practitioners working with teams to take both the individual player and the entire team into account, when trying to mitigate against potential team collapse. On an individual level, similar interventions to those that have been shown to be effective for individual choking can be used to educate players of a team about self-regulation strategies to stabilize performance. Hereby, strategies involving individual attention regulation may be of limited benefit, as many aim at gaining back an automated movement, which is not helpful in regular game situations in team sport (except for penalty shootings etc.). Thus, the major focus should be based on techniques aiming at a change of cognitive appraisals and those involving arousal regulation, because being able to change perspective and evaluate the situation differently as well as being able to downregulate one’s own negative affect is a first step in recovering oneself prior to helping teammates and in prohibiting a transfer of the own affect to others (Tamminen and Crocker, 2013).

On a team level, several different strategies might be considered. Firstly, it is important to make a team aware of the possibility of a team collapse and prepare them for the occurrence of such an event (Wergin et al., 2018, 2019). As a specific strategy of preparation, a solution focused approach developed by Maechel et al. (2021) on the principles of athlete leadership, may be applied. The team development program aims at improving a team’s performance through different processes. Initially, a team is asked about what is going well in their team from their perception, before working out which processes could be improved. In a final step, the team develops and agrees on specific steps to be taken, in order to reach their goals for improvement in a democratic and empowering process. This tool can easily be adapted to prepare a team for a collective team collapse situation. The players would have to reflect on what they did well in a team collapse situation of the past, what they could to better, and how they want to achieve this in a future team collapse situation.

Secondly, teams should work on emotion regulation strategies, as emotion regulation has been shown to improve team performance (Tamminen and Crocker, 2013). Since the development of interpersonal emotion regulation strategies is still in its infancy, teams could focus on basic implementations, such as, discussing their individual preferences for interactions with teammates when facing serious underperformance situations. If teammates know how to motivate and emotionally and socially support each other without taking the risk of escalating negative emotions, team members already have tools to conquer the emotional mechanisms of team collapse. Thus, team communication training could be an important tool to stabilize performance promoting adaptive affect within a team.

Thirdly, as a team collapse event is often accompanied by a decrease of communication in the team, interventions could focus on maintaining the quality and quantity of communication in difficult game situations (Wergin et al., 2018). Certain players could, for example, take responsibility to communicate changes of play or new strategies during team collapse events. Such roles and responsibilities should be determined well ahead of the game or even the season. A discussion about responsibilities and tasks can also be combined with the previously described preparation strategies of Maechel et al. (2021) or discussions about emotion regulation preferences.

Lastly, research has shown that a team culture, in which teammates are blamed for failure, decreases team cohesion and is of disadvantage in difficult game situations (e.g., Wergin et al., 2018, 2019), while a culture of no blame is typically related to team resilience (Morgan et al., 2013). Specific rules of behavior among each other should be mutually developed by team members and fostered by coaches and practitioners. As in modern business companies, a supporting team culture with appreciation as core value and emphasis on individual ownership should be lived. This would also apply to athletes in individual sports.

## Summary and Outlook

Choking under pressure and collective sport team collapse are two distinct constructs explaining performance failure on different levels, which share certain characteristics, but also differ considerably. Team collapse includes many dynamic interpersonal processes, such as the transfer of negative affect between team members or negative behaviors directed at each other, which again impact the further course of the team collapse. These social processes can be seen as key points for the development of interventions that breach the dynamics of a team collapse and therefore recover a team’s performance. It can be assumed that these factors also affect performance in individual sports to some degree and should, therefore, be taken into account in the prevention of choking under pressure in these sports.

Research on choking as well as on team collapse should embrace the development and especially the testing of intervention strategies. While many interventions in the area of choking exist, not all of the interventions used by practitioners have been investigated scientifically. New interventions should view choking as a process and target not only the prevention of the specific choke but also dealing with failure to prevent further chokes and a negative downward spiral over the course of a game or tournament. The development of team collapse interventions is still in its infancy and many more studies on the construct as well as on prevention and intervention strategies are needed.

## Author Contributions

VW wrote the first draft of the manuscript. CM and JB wrote sections of the manuscript. All authors contributed to manuscript revision, read, and approved the submitted version.

## Conflict of Interest

The authors declare that the research was conducted in the absence of any commercial or financial relationships that could be construed as a potential conflict of interest.

## Publisher’s Note

All claims expressed in this article are solely those of the authors and do not necessarily represent those of their affiliated organizations, or those of the publisher, the editors and the reviewers. Any product that may be evaluated in this article, or claim that may be made by its manufacturer, is not guaranteed or endorsed by the publisher.

## References

[B1] Apitzsch E. (2006). Collective collapse in team sports: a theoretical approach. *Curr. Res. Top. Exerc. Sport Psychol. Eur.* 3 35–46.

[B2] Balk Y. A. Adriaanse M. A. De Ridder D. T. Evers C. (2013). Coping under pressure: employing emotion regulation strategies to enhance performance under pressure. *J. Sport Exerc. Psychol.* 35 408–418. 10.1123/jsep.35.4.408 23966450

[B3] Barsade S. (2002). The ripple effect: emotional contagion and its influence on group behavior. *Administr. Sci. Q.* 47 644–675. 10.2307/3094912

[B4] Baumeister R. F. (1984). Choking under pressure: self-consciousness and paradoxical effects of incentives on skillful performance. *J. Pers. Soc. Psychol.* 46 610–620. 10.1037//0022-3514.46.3.610 6707866

[B5] Beckmann J. Fimpel L. Wergin V. V. (2021). Preventing a loss of accuracy of the tennis serve under pressure. *PLoS One* 16:e0255060. 10.1371/journal.pone.0255060 34310638 PMC8312934

[B6] Beckmann J. Gröpel P. Ehrlenspiel F. (2013). Preventing motor skill failure through hemisphere-specific priming: cases from choking under pressure. *J. Exp. Psychol. Gen.* 142 679–691. 10.1037/a0029852 22946898

[B7] Beilock S. L. Carr T. H. (2001). On the fragility of skilled performance: what governs choking under pressure? *J. Exp. Psychol. Gen.* 130 701–725. 10.1037/0096-3445.130.4.701 11757876

[B8] Buszard T. Farrow D. Masters R. S. W. (2013). What is the ‘significance’ of choking in sport? A commentary on Mesagno and Hill. *Int. J. Sport Psychol.* 44 278–280.

[B9] Cross-Villasana F. Gröpel P. Doppelmayr M. Beckmann J. (2015). Unilateral left-hand contractions produce widespread depression of cortical activity after their execution. *PLoS One* 10:e0145867. 10.1371/journal.pone.0145867 26709832 PMC4692494

[B10] Ehrlenspiel F. Mesagno C. (2021). “Anxiety in sport,” in *Sportpsychologie*, eds Hänsel F. Baumgärtner S. D. Kornmann J. Ennigkeit F. (Berlin: Springer), 267–306.

[B11] Englert C. Oudejans R. R. (2014). Is choking under pressure a consequence of skill-focus or increased distractibility? Results from a tennis serve task. *Psychology* 5 1035–1043. 10.4236/psych.2014.59116

[B12] Eysenck M. W. Calvo M. G. (1992). Anxiety and performance: the processing efficiency theory. *Cogn. Emotion* 6 409–434. 10.1080/02699939208409696

[B13] Eysenck M. W. Derakshan N. Santos R. Calvo M. G. (2007). Anxiety and cognitive performance: attentional control theory. *Emotion* 7 336–353. 10.1037/1528-3542.7.2.336 17516812

[B14] Filho E. (2021). Total team collapse in the 2014 FIFA World Cup semi-final (Brazil 1 – Germany 7): implications for coaching and sport psychology practice. *Int. J. Sport Psychol.* 52 1–10.

[B15] Fletcher D. Sarkar M. (2016). Mental fortitude training: an evidence-based approach to developing psychological resilience for sustained success. *J. Sport Psychol. Action* 7 135–157. 10.1080/21520704.2016.1255496

[B16] Galanis E. Hatzigeorgiadis A. Comoutos N. Papaioannou A. Morres I. D. Theodorakis Y. (2021). Effects of a strategic self-talk intervention on attention functions. *Int. J. Sport Exerc. Psychol.* [Epub ahead of print].

[B17] Gallagher S. (2005). Dynamic models of body schematic processes. *Adv. Conscious. Res.* 62:233. 10.1075/aicr.62.15gal 33486653

[B18] Goldenberg A. Halperin E. van Zomeren M. Gross J. (2016). The process model of group-based emotion: integrating intergroup emotion and emotion regulation perspectives. *Pers. Soc. Psychol. Rev.* 20 118–141. 10.1177/1088868315581263 25870386

[B19] Gross J. J. (2002). Emotion regulation: affective, cognitive, and social consequences. *Psychophysiology* 39 281–291. 10.1017/s0048577201393198 12212647

[B20] Gross J. J. (2015). The extended process model of emotion regulation: elaborations, applications, and future directions. *Psychol. Inq.* 26 130–137. 10.1080/1047840X.2015.989751

[B21] Gucciardi D. F. Longbottom J. L. Jackson B. Dimmock J. A. (2010). Experienced golfers’ perspectives on choking under pressure. *J. Sport Exerc. Psychol.* 32 61–83. 10.1123/jsep.32.1.61 20167952

[B22] Haehl W. Mirifar A. Beckmann J. (2022). Regulate to facilitate: a scoping review of prefrontal asymmetry in sport and exercise. *Psychol. Sport Exerc.* 60:102143.

[B23] Hatfield E. Cacioppo J. Rapson R. L. (1994). *Emotional Contagion.* New York, NY: Cambridge University Press. 10.1017/CBO9781139174138

[B24] Hill D. M. Hanton S. Matthews N. Fleming S. (2011). Alleviation of choking under pressure in elite golf: an action research study. *Sport Psychol.* 25 465–488. 10.1123/tsp.25.4.465

[B25] Hill D. M. Shaw G. (2013). A qualitative examination of choking under pressure in team sport. *Psychol. Sport Exerc.* 14 103–110. 10.1016/j.psychsport.2012.07.008

[B26] Jackson R. C. (2013). Babies and bathwater: commentary on Mesagno and Hill’s proposed re-definition of ‘choking’. *Int. J. Sport Psychol.* 44 281–284.

[B27] Jermann F. Van der Linden M. d’Acremont M. Zermatten A. (2006). Cognitive emotion regulation questionnaire (CERQ). *Eur. J. Psychol. Assess.* 22 126–131.

[B28] Kelly J. R. Barsade S. G. (2001). Mood and emotions in small groups and work teams. *Organ. Behav. Hum. Decision Process.* 86 99–130. 10.1006/obhd.2001.2974

[B29] Kleine D. (1990). Anxiety and sport performance: a meta-analysis. *Anxiety Res.* 2 113–131. 10.1080/08917779008249330

[B30] Krawietz S. A. (2013). *Alleviating Choking Under Pressure Using Imagery.* Ann Arbor, MI: ProQuest Dissertations Publishing.

[B31] Lam W. K. Maxwell J. P. Masters R. S. W. (2009). Analogy versus explicit learning of a modified basketball shooting task: performance and kinematic outcomes. *J. Sports Sci.* 27 179–191. 10.1080/02640410802448764 19153868

[B32] Leary M. R. (1992). Self-presentational processes in exercise and sport. *J. Sport Exerc. Psychol.* 14 339–335. 10.1111/josh.12756 30916416

[B33] Leary M. R. Kowalski R. M. (1990). Impression management: a literature review and two-component model. *Psychol. Bull.* 107 34–47. 10.1037/0033-2909.107.1.34

[B34] Liao C. M. Masters R. S. (2001). Analogy learning: a means to implicit motor learning. *J. Sports Sci.* 19 307–319. 10.1080/02640410152006081 11354610

[B35] Maechel C. Loughead T. M. Wergin V. V. Kossak T. Beckmann J. (2021). A solution-focused approach to shared athlete leadership development using mixed methods. *Sport Psychol.* 1 1–14.

[B36] Masters R. S. (2000). Theoretical aspects of implicit learning in sport. *Int. J. Sport Psychol.* 31 530–541. 10.7717/peerj.11289 33954060 PMC8053378

[B37] Masters R. S. W. (1992). Knowledge, knerves and know-how: the role of explicit versus implicit knowledge in the breakdown of a complex motor skill under pressure. *Br. J. Psychol.* 83 343–358. 10.1016/j.concog.2007.03.009 17470398

[B38] McRae K. Gross J. J. (2020). Emotion regulation. *Emotion* 20 1–9. 10.1037/emo0000703 31961170

[B39] Mesagno C. Beckmann J. (2017). Choking under pressure: theoretical models and interventions. *Curr. Opin. Psychol.* 16 170–175. 10.1016/j.copsyc.2017.05.015 28813345

[B40] Mesagno C. Beckmann J. Wergin V. V. Gröpel P. (2019). Primed to perform: comparing different pre-performance routine interventions to improve accuracy in closed, self-paced motor tasks. *Psychol. Sport Exerc.* 43 73–81. 10.1016/j.psychsport.2019.01.001

[B41] Mesagno C. Geukes K. Larkin P. (2015). “Choking under pressure: a review of current debates, literature, and interventions,” in *Contemporary Advances in Sport Psychology: A Review*, eds Mellalieu S. Hanton S. (London: Routledge), 148–174.

[B42] Mesagno C. Harvey J. T. Janelle C. M. (2011). Self-presentation origins of choking: evidence from separate pressure manipulations. *J. Sport Exerc. Psychol.* 33 441–459. 10.1123/jsep.33.3.441 21659672

[B43] Mesagno C. Hill D. (2013). Definition of choking in sport: re-conceptualization and debate. *Int. J. Sport Psychol.* 44 267–277.

[B44] Mesagno C. Marchant D. Morris T. (2009). Alleviating choking: the sounds of distraction. *J. Appl. Sport Psychol.* 21 131–147. 10.1080/10413200902795091

[B45] Mesagno C. Mullane-Grant T. (2010). A comparison of different pre-performance routines as possible choking interventions. *J. Appl. Sport Psychol.* 22 343–360. 10.1080/10413200.2010.491780

[B46] Moll T. Jordet G. Pepping G. J. (2010). Emotional contagion in soccer penalty shootouts: celebration of individual success is associated with ultimate team success. *J. Sports Sci.* 28 983–992. 10.1080/02640414.2010.484068 20544488

[B47] Morgan P. B. Fletcher D. Sarkar M. (2013). Defining and characterizing team resilience in elite sport. *Psychol. Sport Exerc.* 14 549–559.

[B48] Muraven M. Tice D. Baumeister R. (1998). Selfcontrol as a limited resource: regulatory depletion patterns. *J. Pers. Soc. Psychol.* 74 774–789. 10.1037/0022-3514.74.3.774 9523419

[B49] Niven K. (2017). The four key characteristics of interpersonal emotion regulation. *Curr. Opin. Psychol.* 17 89–93. 10.1016/j.copsyc.2017.06.015 28950980

[B50] Oudejans R. R. Pijpers J. R. (2009). Training with anxiety has a positive effect on expert perceptual–motor performance under pressure. *Q. J. Exp. Psychol.* 62 1631–1647. 10.1080/17470210802557702 19123115

[B51] Oudejans R. R. Pijpers J. R. (2010). Training with mild anxiety may prevent choking under higher levels of anxiety. *Psychol. Sport Exerc.* 11 44–50. 10.1016/j.psychsport.2009.05.002

[B52] Oudejans R. R. D. Kuijpers W. Kooijman C. C. Bakker F. C. (2011). Thoughts and attention of athletes under pressure: skill-focus or performance worries? *Anxiety Stress Coping* 24 59–73. 10.1080/10615806.2010.481331 20425657

[B53] Parnabas V. A. Mahamood Y. Parnabas J. Abdullah N. M. (2014). The relationship between relaxation techniques and sport performance. *Univ. J. Psychol.* 2 108–112.

[B54] Pates J. Karageorghis C. I. Fryer R. Maynard I. (2003). Effects of asynchronous music on flow states and shooting performance among netball players. *Psychol. Sport Exerc.* 4 415–427.

[B55] Pollakoff B. (2015). *Dirk Nowitzki Used to Sing a Counting Crows Song at the Free Throw Line to Relax COMPLEX.* Available online at: https://www.complex.com/sports/2015/08/dirk-nowitzki-counting-crows-song-free-throw-line (accessed March 05, 2022).

[B56] Prapavessis H. Grove J. R. Eklund R. C. (2004). Self-presentational issues in competition and sport. *J. Appl. Sport Psychol.* 16 19–40. 10.1016/j.ptsp.2010.07.005 21256448

[B57] Schmeichel B. J. Vohs K. D. Baumeister R. F. (2003). Intellectual performance and ego depletion: role of the self in logical reasoning and other information processing. *J. Pers. Soc. Psychol.* 85 33–46. 10.1037/0022-3514.85.1.33 12872883

[B58] Scott-Hamilton J. Schutte N. S. Brown R. F. (2016). Effects of a mindfulness intervention on sports-anxiety, pessimism, and flow in competitive cyclists. *Appl. Psychol. Health Well Being* 8 85–103. 10.1111/aphw.12063 26970111

[B59] Siegel A. (2011). *The 10 Worst Collapses in Sports. USA Today Sports.* Available online at: https://ftw.usatoday.com/2016/04/jordan-spieth-sports-collapses (accessed June 6, 2022).

[B60] Smith R. E. Smoll F. L. (1990). “Sport performance anxiety,” in *Handbook of Social and Evaluation Anxiety*, ed. Leitenberg H. (Boston, MA: Springer), 417–454. 10.1007/978-1-4899-2504-6_14

[B61] Tajfel H. (1978). “The achievement of inter-group differentiation,” in *Differentiation Between Social Groups*, ed. Tajfel H. (London: Academic Press), 77–100.

[B62] Tajfel H. Turner J. C. (1979). An integrative theory of intergroup conflict. *See Austin Worchel* 1979 33–47.

[B63] Tamminen K. A. Crocker P. R. (2013). “I control my own emotions for the sake of the team”: emotional self-regulation and interpersonal emotion regulation among female high-performance curlers. *Psychol. Sport Exerc.* 14 737–747. 10.1016/j.psychsport.2013.05.002

[B64] Tamminen K. A. Gaudreau P. McEwen C. E. Crocker P. R. E. (2016a). Interpersonal emotion regulation among adolescent athletes: a Bayesian multilevel model predicting sport enjoyment and commitment. *J. Sport Exerc. Psychol.* 38 541–555. 10.1123/jsep.2015-0189 27383379

[B65] Tamminen K. A. Palmateer T. M. Denton M. Sabiston C. Crocker P. R. Eys M. (2016b). Exploring emotions as social phenomena among Canadian varsity athletes. *Psychol. Sport Exerc.* 27 28–38. 10.1016/j.psychsport.2016.07.010

[B66] Tamminen K. A. Kim J. Danyluck C. McEwen C. E. Wagstaff C. R. Wolf S. A. (2021). The effect of self-and interpersonal emotion regulation on athletes’ anxiety and goal achievement in competition. *Psychol. Sport Exerc.* 57: 102034.

[B67] Tamminen K. A. Page-Gould E. Schellenberg B. Palmateer T. Thai S. Sabiston C. M. (2019). A daily diary study of interpersonal emotion regulation, the social environment, and team performance among university athletes. *Psychol. Sport Exerc.* 45:101566. 10.1016/j.psychsport.2019.101566

[B68] Totterdell P. (2000). Catching moods and hitting runs: mood linkage and subjective performance in professional sports teams. *J. Appl. Psychol.* 83 848–859. 10.1037/0021-9010.85.6.848 11125650

[B69] Vickers J. N. (2007). *Perception, Cognition, and Decision Training: The Quiet Eye in Action.* Champaign, IL: Human Kinetics.

[B70] von Scheve C. Ismer S. (2013). Towards a theory of collective emotions. *Emot. Rev.* 5 406–413. 10.1177/1754073913484170

[B71] Wagstaff C. R. (2014). Emotion regulation and sport performance. *J. Sport Exerc. Psychol.* 36 401–412. 10.1123/jsep.2013-0257 25226609

[B72] Wallace H. M. Baumeister R. F. Vohs K. D. (2005). Audience support and choking under pressure: a home disadvantage? *J. Sports Sci.* 23 429–438. 10.1080/02640410400021666 16089187

[B73] Wang J. Marchant D. Morris T. Gibbs P. (2004). Self-consciousness and trait anxiety as predictors of choking in sport. *J. Sci. Med. Sport* 7 174–185. 10.1016/s1440-2440(04)80007-0 15362313

[B74] Wergin V. V. Mallett C. J. Mesagno C. Zimanyi Z. Beckmann J. (2019). When you watch your team fall apart–coaches’ and sport psychologists’ perceptions on causes of collective sport team collapse. *Front. Psychol.* 10:1331. 10.3389/fpsyg.2019.01331 31244730 PMC6581729

[B75] Wergin V. V. Zimanyi Z. Beckmann J. (2020). A field study investigating running distance and affect of field hockey players in collective team collapse situations. *Int. J. Sport Exerc. Psychol.* 19 584–597. 10.1080/1612197X.2020.1817123

[B76] Wergin V. V. Zimanyi Z. Mesagno C. Beckmann J. (2018). When suddenly nothing works anymore within a team–Causes of collective sport team collapse. *Front. Psychol.* 9:2115. 10.3389/fpsyg.2018.02115 30459685 PMC6232390

[B77] Wilson P. Eklund R. C. (1998). The relationship between competitive anxiety and self-presentational concerns. *J. Sport Exerc. Psychol.* 20 81–97. 10.1123/jsep.30.3.383 18648111

